# 3D Surface Scanning—A Novel Protocol to Characterize Virtual Nickel–Titanium Endodontic Instruments

**DOI:** 10.3390/ma16103636

**Published:** 2023-05-10

**Authors:** Jorge N. R. Martins, Ricardo Pinto, Emmanuel J. N. L. Silva, Marco Simões-Carvalho, Duarte Marques, Rui F. Martins, Marco A. Versiani

**Affiliations:** 1 Faculdade de Medicina Dentária, Universidade de Lisboa, 1600-277 Lisboa, Portugal; 2 Grupo de Investigação em Bioquimica e Biologia Oral, Unidade de Investigação em Ciências Orais e Biomédicas (UICOB), 1600-277 Lisboa, Portugal; 3 Centro de Estudo de Medicina Dentária Baseada na Evidência (CEMDBE), 1600-277 Lisboa, Portugal; 4 Department of Endodontics, School of Dentistry, Grande Rio University (UNIGRANRIO), Rio de Janeiro 21210-623, Brazil; 5 Department of Endodontics, Fluminense Federal University, Niterio, Rio de Janeiro 24220-900, Brazil; 6 LIBPhys-FCT UID/FIS/04559/2013, 1600-277 Lisboa, Portugal; 7 UNIDEMI, Department of Mechanical and Industrial Engineering, NOVA School of Science and Technology, Universidade NOVA de Lisboa, 2829-516 Caparica, Portugal; 8 Dental Specialty Center, Brazilian Military Police, Belo Horizonte, Minas Gerais 30350-190, Brazil

**Keywords:** 3D imaging, dental instruments, endodontics, Finite Element Analysis, micro-CT, optical scanner, scanning electron microscopy, root canal therapy, virtual model

## Abstract

The nickel–titanium (NiTi) instruments’ geometry plays an important role in their performance and behavior. The present assessment intends to validate and test the applicability of a 3D surface scanning method using a high-resolution laboratory-based optical scanner to create reliable virtual models of NiTi instruments. Sixteen instruments were scanned using a 12-megapixel optical 3D scanner, and methodological validation was performed by comparing quantitative and qualitative measurements of specific dimensions and identifying some geometric features of the 3D models with images obtained through scanning electron microscopy. Additionally, the reproducibility of the method was assessed by calculating 2D and 3D parameters of three different instruments twice. The quality of the 3D models created by two different optical scanners and a micro-CT device was compared. The 3D surface scanning method using the high-resolution laboratory-based optical scanner allowed for the creation of reliable and precise virtual models of different NiTi instruments with discrepancies varying from 0.0002 to 0.0182 mm. The reproducibility of measurements performed with this method was high, and the acquired virtual models were adequate for use in in silico experiments, as well as for commercial or educational purposes. The quality of the 3D model obtained using the high-resolution optical scanner was superior to that acquired by micro-CT technology. The ability to superimpose virtual models of scanned instruments and apply them in Finite Element Analysis and educational purposes was also demonstrated.

## 1. Introduction

A recent bibliometric analysis has highlighted that research on nickel–titanium (NiTi) instruments has been one of the most explored themes in high impact endodontic journals over the past two decades [1]. This research encompasses a broad range of methodologies, from basic in vitro tests examining mechanical resistance to understand behavior under stress [2,3] to more complex approaches using non-destructive imaging systems to compare shaping ability on real root canals regarding several parameters, such as unprepared walls, volume of removed dentine, bacterial reduction, extruded debris, transportation, and microcrack formation [4]. Other methods have also been applied in the study of NiTi instruments, including analyses of cutting efficiency [5], surface roughness [6], gutta-percha removal [7], fracture surface [8], and incidence of fracture [9], among others. The findings of these studies are primarily associated with the intrinsic factors of the instruments, including the type of heat treatment applied during manufacturing and the design [3]. Overall, the comprehensive research on NiTi instruments has significantly contributed to our understanding of their behavior, and these findings may have important implications for improving their clinical performance.

The impact of NiTi instrument geometry on mechanical efficiency has been extensively debated in the literature [10]. However, most studies assessing instrument design have relied on information provided by manufacturers or the analyzing cross-sections and surface features [11], using stereomicroscopy [10] or scanning electron microscopy (SEM) [11]. More recently, attempts have been made to create 3D models of NiTi instruments using the non-destructive micro-CT technology [12,13]. However, these studies have shown that virtual models produced exhibit significant changes in design, including severely flattened cutting blades and deformations in the geometry of their tips. These findings suggest that micro-CT may not be the most appropriate method for reproducing fine details of NiTi instruments. Therefore, there is a need to develop alternative methods for creating precise and accurate 3D models of NiTi instruments that can capture their intricate design features and be used for further research and applications in the field of endodontics.

The existing techniques for evaluating the design of endodontic instruments have inherent limitations that either require partial or full destruction of samples or provide only a two-dimensional image of a 3D structure. As a result, there is a need for a non-destructive method that can create precise 3D models of NiTi instruments for in silico research. This exploratory study presents a novel method for creating reliable virtual models of NiTi instruments using a high-resolution laboratory-based 3D surface optical scanner. In order to answer the hypothesis of whether this method is accurate and reproducible, the method is validated through qualitative and quantitative comparisons with a gold standard method and well-known measurements, as well as through testing the reproducibility of measurements. A qualitative comparison of 3D models created by two different laboratory-based optical scanners and a micro-CT device is also performed. The potential applications of this method are demonstrated, including its use in Finite Element Analysis and for commercial or educational purposes.

## 2. Materials and Methods

Sixteen 25 mm NiTi instruments were used in this study including 2 ProTaper Gold S1 (Dentsply Sirona, Ballaigues, Switzerland; LOT 1725638); 1 ProGlider (Dentsply Sirona, Ballaigues, Switzerland; LOT 1526881); 3 ProTaper Next X2 (Dentsply Sirona, Ballaigues, Switzerland; LOT 1784995); 1 EdgeOne Fire Primary (EdgeEndo, Johnson City, TN, USA; LOT 121719026); 5 Reciproc R25 (VDW, Munich, Germany; LOT 396896); 2 ProFile size 25, taper 0.06 (Dentsply Tulsa, Tulsa, OK, USA; LOT 1784996); 1 ProTaper Gold F2 (Dentsply Sirona, Ballaigues, Switzerland; LOT 1706721); and 1 ProTaper Universal F3 (Dentsply Maillefer, Ballaigues, Switzerland; LOT 4574420).

### 2.1. 3D Surface Scanning Procedure

**Figure 1:**
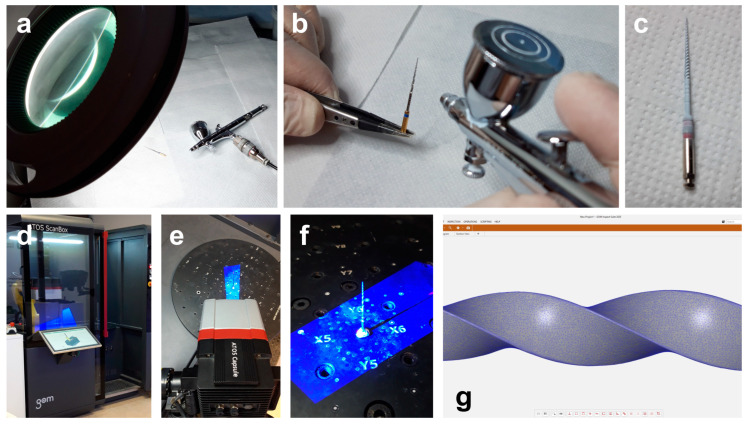
Procedural steps of the 3D surface scanning method: (**a**) Loupe magnification and precision airbrush gun; (**b**) Application of the anti-reflective coating spray on the surface of the NiTi instrument; (**c**) NiTi rotary instrument covered by the anti-reflective coating; (**d**) Scanbox of the 12 MP laboratory-based optical scanner; (**e**) Laboratory-based scanner head during the scanning procedure; (**f**) Instrument mounted in the object holder during the 360° scanning process; (**g**) Virtual 3D model of the scanned instrument showing its triangulated mesh.

The high-resolution optical 3D scanner (ATOS 3D scanner; GOM, Braunschweig, Germany) mounted with an ATOS capsule 12 M MV40 (GOM, Braunschweig, Germany) was used to scan the selected instruments’ surfaces. To minimize scan noise and surface gloss, a 2 µm thick layer of an anti-reflective coating spray was manually applied on the instruments’ surface under loupe magnification (×3.5), using a precision airbrush with flow control (Figure 1a–c). Each instrument was then mounted in the scanbox object holder (ATOS Scanbox 4105; GOM, Braunschweig, Germany) and scanned at resolution of 12 megapixels (MP) in 24 positions through 360°, with a working distance of 290 mm (Figure 1d–f). A dedicated software (GOM Inspect Suite 2020; GOM, Braunschweig, Germany) was used to automatically obtain a triangulated mesh in STL format from the points cloud (Figure 1g).

### 2.2. Qualitative and Quantitative Validations

**Figure 2:**
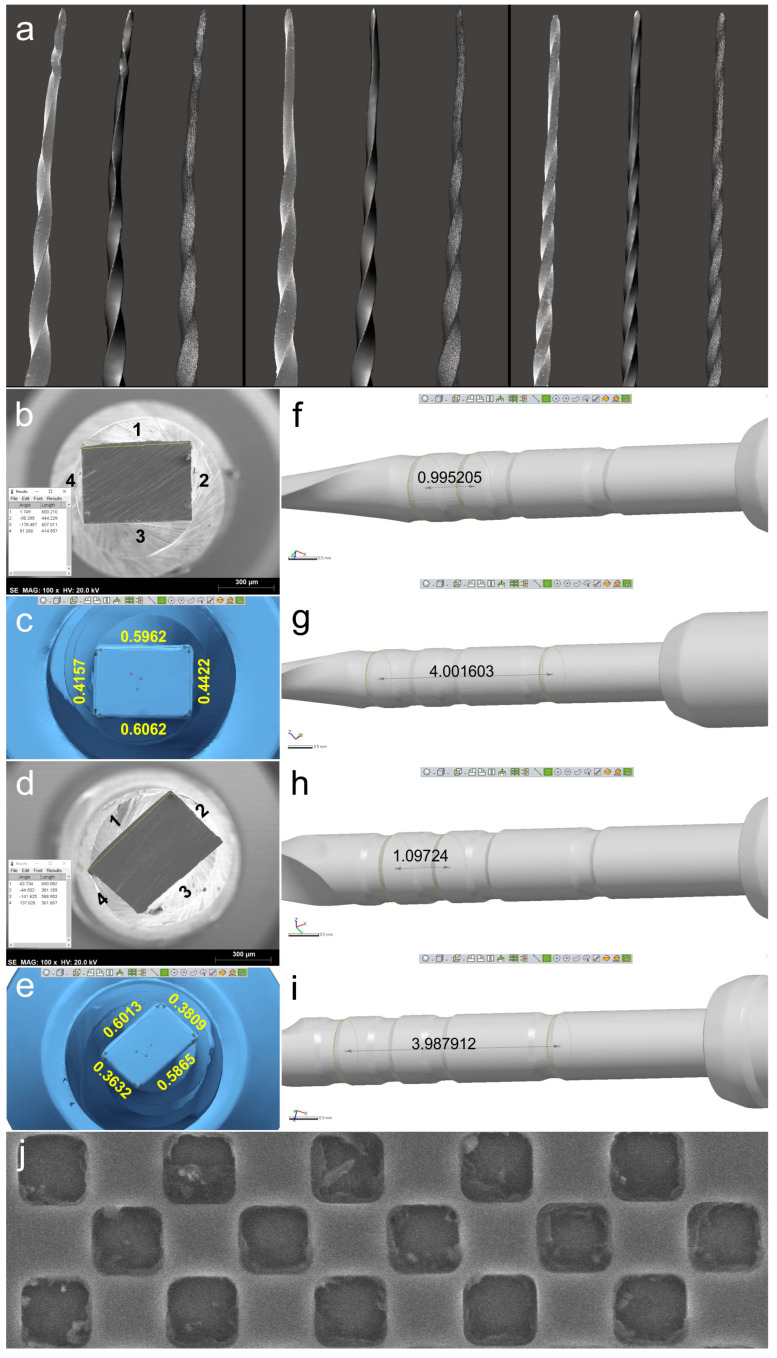
Qualitative and quantitative validations: (**a**) SEM image of each deformed instrument (on the left) was visually compared to the virtual 3D model created by the scanning procedure (in the center). On the right, it is possible to observe the polygonal mesh of each virtual model; (**b**–**e**) Sides of ProTaper Next X2 (**b**) and EdgeOne Fire Primary (**d**) cross-sections analyzed by SEM (**b**,**d**) and by the 3D surface scanning method (**c**,**e**) showing the similarity of results; (**f**–**i**) Distances were calculated in the virtual models regarding the measuring lines references from 18 mm and 19 mm (**f**,**h**) and from 18 mm and 22 mm (**g**,**i**) of ProTaper Next X2 and EdgeOne Fire Primary instruments, demonstrating they were similar to known lengths reported by the manufacturers. Representative image of the SEM calibration grid (**j**).

The objective of the qualitative validation was to assess the ability of the 3D surface scanning method to capture and reproduce intricate geometric features of NiTi instruments. To accomplish this, three small-sized rotary instruments (2 ProTaper Gold S1 and 1 ProGlider) that had become deformed at the apical 3 mm during clinical use were selected and scanned using the method previously described. The deformations were utilized as a reference to perform a visual comparison, since new instruments retain their structure. Subsequently, the instruments were cleaned in an ultrasonic bath, mounted on a sample holder, and placed within the vacuum chamber of a SEM device (Hitachi S-2400, Hitachi, Tokyo, Japan) equipped with a Bruker Esprit 1.9 (Bruker Quantax, Bruker Corporation, Billerica, MA, USA) operating at 20 kW. Images were captured from their apical 6–7 mm at ×20 magnification and compared qualitatively with their corresponding 3D surface models using MeshLab v2020.02 (ISTI, Pisa, Italy) (Figure 2a).

For the quantitative validation, two NiTi rotary instruments with rectangular cross-sections (1 ProTaper Next X2 and 1 EdgeOne Fire Primary) were compared with SEM images and previously known lengths. The instruments were sectioned perpendicularly to their long axis at a random position in the middle using a diamond disc. After cleaning the samples in an ultrasound bath, the 3D models of the instruments were obtained using the optical 3D scanner. Then, SEM analyses were conducted and cross-sections were assessed at ×100 magnification (Ted Pella, Redding, CA, USA).

Images were exported in TIFF format with the digital scale after checking the magnification scale using a standard 300 µm calibration grid (Ted Pella, Redding, CA, USA). The lengths of the 4 sides of each rectangular cross-section were measured using the straight segment tool of the ImageJ software (Laboratory for Optical and Computational Instrumentation, Madison, WI, USA) after calibrating with the SEM digital scale (Figure 2b,d).

In Geomagic Control X software (3D Systems, Morrisville, NC, USA), the 3D models in STL format were aligned with the SEM images. Each vertex of the rectangular cross-section was digitally identified and each side was measured using the Linear Dimension tool (Figure 2c,e). The Shapiro–Wilk test was used to evaluate the normality of variance of the 8 side lengths obtained, and the Mann–Whitney test (non-Gaussian distribution) was used to compare the SEM and 3D scanner groups, with a significance level set at 5% (SPSS v22.0 for Windows; SPSS Inc., Chicago, IL, USA). The distances between the measuring lines, positioned at the non-cutting blade area, were measured in millimeters (Geomagic Control, 3D Systems; Morrisville, NC, USA) and compared to known standard values provided by the manufacturers (Figure 2f–i).

### 2.3. Reproducibility of Measurements

**Figure 3:**
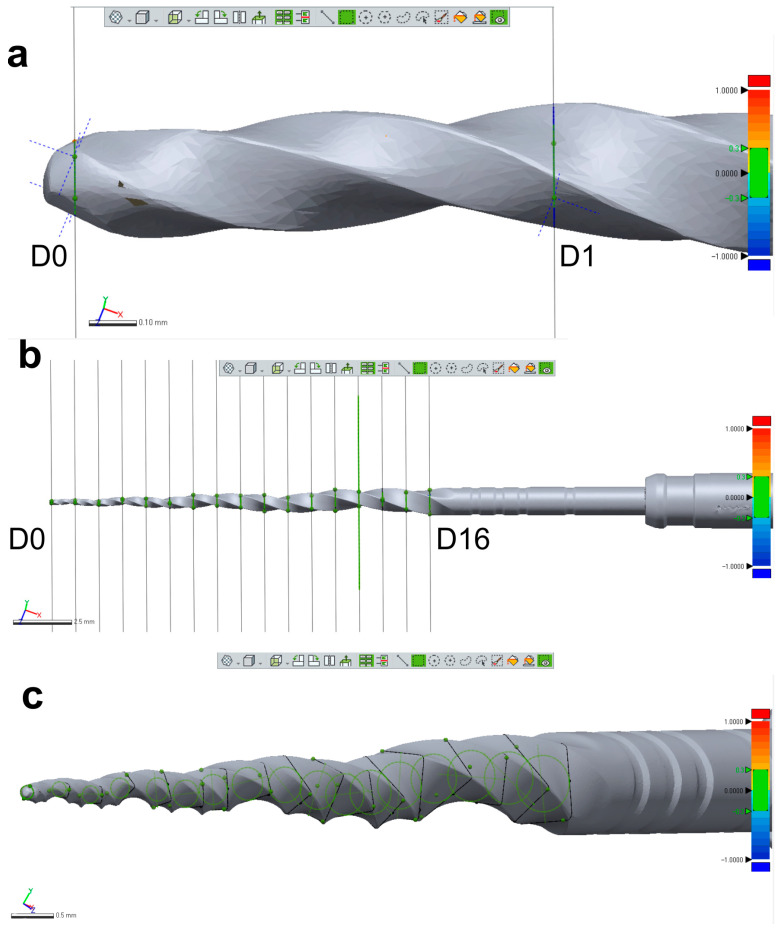
Measurement assessments conducted on a ProTaper Next X2 instrument: (**a**) D0 was set at the base of instrument’s tip; (**b**) A total of 17 cross-section levels were established from D0 to D16; (**c**) Multiple measurements were conducted of each level showed in (**b**).

Three new 25 mm NiTi instruments with different designs (Reciproc R25; ProFile size 25, taper 0.06; and ProTaper Next X2) were selected for 3D surface scanning, as previously described. In each 3D model, a virtual plane was created perpendicularly to the long axis at the base of the handle, and another parallel plane was created near the tip of the instrument with an offset of 25 mm, and adjusted to match the D0 level (Geomagic Control X; 3D Systems) (Figure 3a). Subsequently, virtual planes from D1 to D16 of the active blade were obtained with an offset of 1 mm using D0 as a reference (Figure 3b). The cross-sections created by these 17 virtual planes (positioned from D0 to D16) were analyzed regarding 2D parameters ((perimeter (mm), area (mm^2^), long axis (mm), and core diameter (mm)), while 3D parameters ((surface area (mm^2^) and volume (mm^3^)) of the whole active blade (from D0 to D16) were also calculated (Figure 3c). To determine the reproducibility of the method, the measurements of 3D and 2D parameters at D1, D8, and D16 levels were repeated 10 times, and the standard deviation was calculated. Furthermore, the measurements were repeated twice in an 8-week interval in each instrument (to guarantee the observer was not influenced by the initial assessment), and the results were combined to calculate their average. The similarity between both evaluations was assessed using the interclass correlation coefficient test.

### 2.4. Optical Scanners vs. Micro-CT

**Figure 4:**
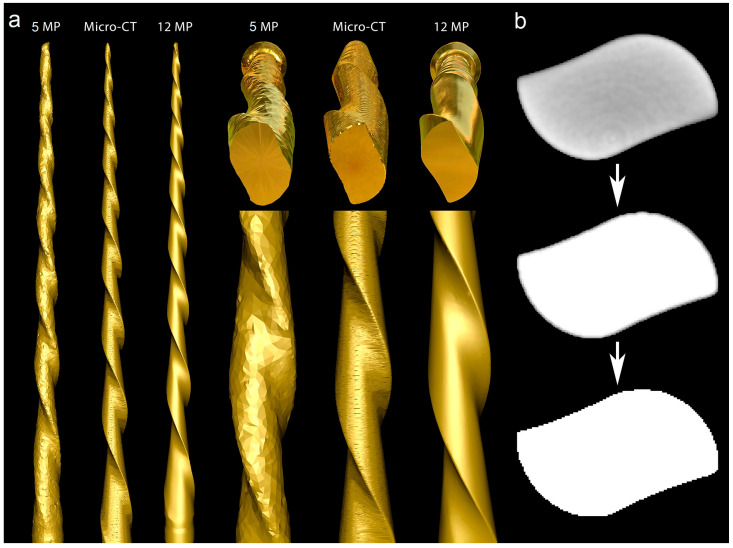
Optical scanners vs. micro-CT: (**a**) The 5 MP scan created a model with rounded blades, no tip, and severe artificial surface irregularities. The surface appearance of the 3D model acquired by the micro-CT scanner showed better quality than the 5 MP resolution scanner, but the generated model had slightly flattened blades and artificial surface irregularities, while the highest quality model was created by the 12 MP optical scanner; (**b**) Cross-section of the Reciproc instrument reconstructed from a micro-CT scan in which irregularities can be observed in its external surface after the binarization process caused by the high density of the metal alloy.

A new 25 mm Reciproc R25 instrument was imaged in a micro-CT scanner (SkyScan 1173; Bruker-microCT, Kontich, Belgium) set at a pixel size of 7.48 µm, 90 kV, 88 µA, 360° rotation with steps of 0.4°, exposure time of 800 ms, and frame averaging of 5, filtered by a 1.0 mm thick aluminum foil. The images were reconstructed using a ring artifact correction of 4, beam hardening correction of 45%, and a contrast limit ranging from 0.10 to 0.30 (NRecon v.1.7.16; Bruker-microCT, Kontich, Belgium), resulting in 3400 grayscale cross-section images of the instrument. The 3D model of the instrument was generated in STL format using CTAn v.1.20.8 software (Bruker-microCT, Kontich, Belgium). Subsequently, the mentioned instrument was subjected to 3D surface scanning in 2 laboratory-based optical scanners with resolutions of 12 MP (ATOS 3D; GOM, Braunschweig, Germany) and 5 MP (S900 ARTI; Zirkonzhan, Gais, Italy) to generate 3D surface models in an STL format. The quality of 3D surface models acquired by both optical scanners was evaluated and compared with the micro-CT model of the instrument using a qualitative approach in MeshLab v2020.02 software (ISTI, Pisa, Italy) (Figure 4).

### 2.5. Research Application: Changes in the Instrument’s Morphology

After obtaining approval from the local Ethics Committee (CE-FMDUL 13-10-20), four mandibular molars with fully formed apices and two independent mesial canals were selected for the study. Conventional access cavities were prepared and followed by establishing apical patency using a size 08 K-file (Dentsply Maillefer, Ballaigues, Switzerland). Glide path was then created using sizes 10 and 15 K-files (Dentsply Maillefer, Ballaigues, Switzerland) up to the working length set 1 mm short of the foramen. The preparation of all canals (n = 8) was performed using a single Reciproc R25, activated by an electric motor (VDW Silver; VDW, Munich, Germany) up to the working length as per the manufacturer’s instructions. Before preparation, a 3D virtual model of the instrument was obtained using the 12 MP resolution optical scanner, as previously described.

**Figure 5:**
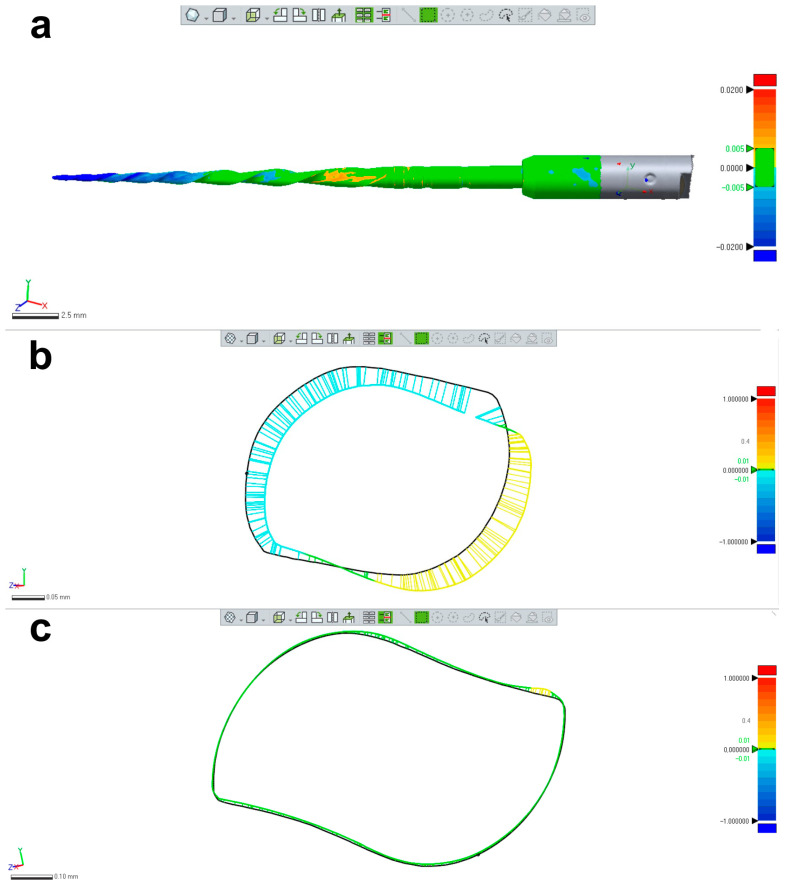
Research application: changes in the instrument’s morphology. (**a**) Superimposition of 2 STL volumes from the same NiTi instrument (Reciproc R25) created before and after preparation of 8 mesial root canals of mandibular molars showing permanent geometric deformation at the apical area; (**b**) Changes in the design of the apical area were mostly due to unwinding; (**c**) In most of the coronal part of the blade no relevant changes were noticed. The black line in (**b**,**c**) represents the contours of the instrument before preparation.

Each canal was irrigated using a 27G needle with a slotted-end tip (Coltene, Langenau, Germany) with 5 mL of 5.25% sodium hypochlorite. An experienced operator, working under magnification (Opmi Pico; Carl Zeiss Surgical, Jena, Germany), shaped the canals using the Reciproc R25 instrument without sterilizing it between the shaping procedures. After shaping, the instrument was cleaned with a compress soaked in alcohol, visually inspected at ×13.6 magnification (Opmi Pico; Carl Zeiss Surgical, Jena, Germany), and rescanned using the 12 MP resolution optical scanner. The resulting STL files were imported into the Geomagic Control X software (3D Systems; Morrisville, NC, USA), where they were superimposed, and the 3D Compare tool was used to evaluate the induced changes in the instrument’s morphology both qualitatively (color-coded from blue to red) and quantitatively (Figure 5).

### 2.6. Research Application: Finite Elements Analysis (FEA)

A 3D model of a Reciproc R25 instrument was generated using the 12 MP resolution optical scanner, as previously described. The SLT model was processed using an open-source software (Blender v.3.3.1; Blender Foundation, Amsterdam, The Netherlands) to correct triangulated mesh defects in its surface, followed by more complex geometry errors using the Analysis-Inspector tool in Meshmixer v.3.5 software (Autodesk Inc., San Rafael, CA, USA). Smoothing was applied to specific areas while preserving geometric details. However, to minimize the impact of increasing the number of triangular elements on the surface of the model, the Quadratic Edge Collapse Decimation functionality of MeshLab software (ISTI, Pisa, Italy) was employed to reduce the number of triangular elements while preserving the original topology of the model. The reduction in the number of triangular elements helps to decrease the discretization and convergence times of the finite element meshes, thus reducing the processing capacity required by the computer. The SLT model was then rechecked for potential geometric errors using the Import Diagnostics and Check Geometry tool in Solidworks software (Dassault Systemes, Waltham, MA, USA). Next, the model was prepared for static simulation of a torsion test using FEA in Solidworks Simulation (Dassault Systèmes, Waltham, MA, USA), where the mesh density (i.e., the number of finite elements that define it), boundary conditions, applied load, and material properties were set. The 3D geometry of finite elements had the shape of a tetrahedra with second-order definition, defined by nodes in their four vertexes and nodes in the middle of their six edges.

**Figure 6:**
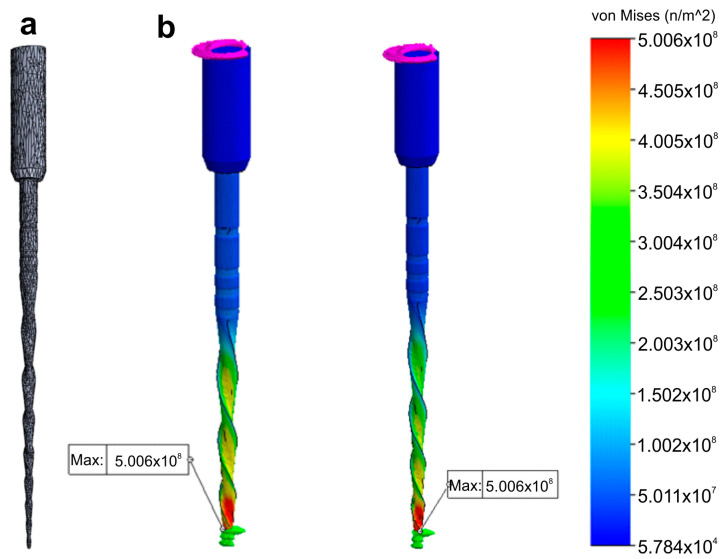
Research application: Finite Elements Analysis. Stress distribution (in MPa) induced in an 3D model of a NiTi instrument created using a high-resolution optical scanner by a torque applied at its tip: (**a**) Polygonal 3D mesh of the surface model; (**b**) Simulated torsion test at 2 different levels of the model.

The model nodes were assigned a certain number of degrees of freedom to account for nodal displacements and rotations, along with material properties that dictate the mechanical response of the model during numerical simulations. Regarding the boundary and loading conditions, a section of the instrument was specified to mimic the instrument lock in the root canal and a torsional moment was applied in the connection zone of the instrument to the motor (Figure 6).

### 2.7. Other Applications: 3D Models for Commercial or Teaching Purposes

**Figure 7:**
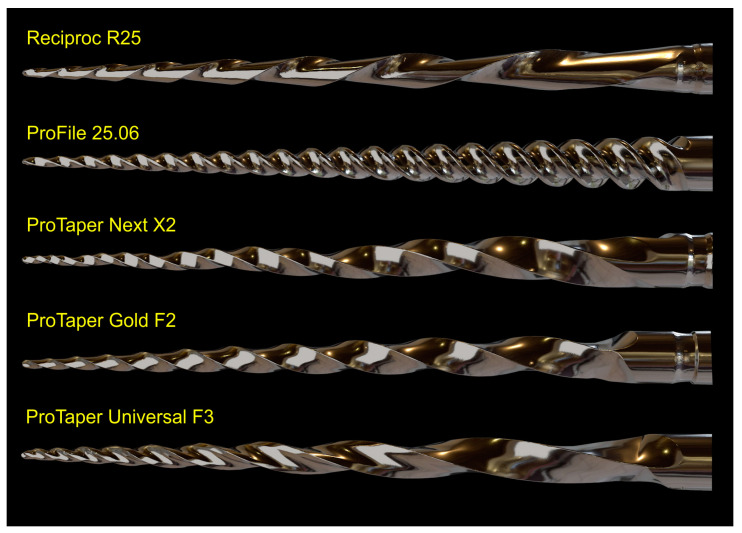
3D models for commercial or teaching purposes. 3D virtual models of 5 NiTi instruments created using the 3D surface scanning method and artificially texturized with a metal shader to simulate real instruments.

Five NiTi instruments (Reciproc R25; ProFile size 25, taper 0.06; ProTaper Next X2; ProTaper Gold F2; and ProTaper Universal F3) were imaged using a 12 MP resolution optical scanner. The resulting 3D virtual models in STL format were imported into Blender v.3.3.1 software (Blender Foundation, Amsterdam, Netherlands) and rendered with a metal shader to simulate the real instruments (Figure 7). Subsequently, dedicated tools were used to create realistic movies demonstrating the instruments’ rotational movement and their application during the preparation of a virtual root canal.

## 3. Results

### 3.1. Qualitative and Quantitative Validations

The 3D surface models were found to be consistent with the SEM images of three permanently deformed rotary NiTi instruments (Figure 2a). In addition, the cross-sectional measurements (in millimeters) based on both SEM images and 3D models of the instruments were compared, revealing only minor differences. Discrepancies observed in the ProTaper Next instrument were 0.5860 vs. 0.5962 (side 1), 0.4300 vs. 0.4422 (side 2), 0.5880 vs. 0.6062 (side 3), and 0.4090 vs. 0.4157 (side 4), while in the EdgeOne Fire they were 0.6030 vs. 0.6013 (side 1), 0.3870 vs. 0.3809 (side 2), 0.5820 vs. 0.5865 (side 3), and 0.3630 vs. 0.3632 (side 4). The lowest and highest differences were observed on sides 4 (0.0067 mm) and 3 (0.0182 mm) of the ProTaper Next, and on sides 4 (0.0002 mm) and 2 (0.0061 mm) of the EdgeOne Fire (Figure 2b–e), but the statistical analysis showed no significant difference between the results obtained from the SEM and 3D scanner (*p* = 0.645). A sample size calculation was performed to determine the required sample size for a statistical power of 80%, an alpha-type error of 0.05, a standard deviation of 0.1028, and an effect size of 0.0442. The resulting sample size of 87 measurements per group was deemed not clinically significant, and thus no further similar evaluations were done. For the ProTaper Next instrument, the measuring lines exhibited differences of 0.0048 mm (between 20 and 21 mm lines) and 0.0016 mm (between 20 and 24 mm lines), while the EdgeOne Fire exhibited differences of 0.0972 mm and 0.0121 mm (Figure 2f–i).

### 3.2. Reproducibility of Measurements

Table 1 and Table 2 summarize the 2D and 3D measurements conducted on virtual models of three distinct NiTi instruments from D0 to D16. The standard deviations obtained from repeated measurements of 2D parameters at D1, D8, and D16 levels (Table 1), as well as the volume and surface area (Table 2), were extremely low. The interclass correlation coefficient test demonstrated values of 0.999 and 1.000 when comparing the results of twice-measured 2D parameters within an 8-week interval.

### 3.3. Optical Scanners vs. Micro-CT

The 12 MP optical scanning method provided the most accurate virtual reproduction of the NiTi instrument design. In contrast, the 5 MP resolution optical scanner failed to replicate the instrument’s overall geometry, including its cutting blade edges, surface, and tip geometry. Although the micro-CT scanner produced a 3D model with better surface quality than the 5 MP scanner, there were still irregularities, such as external surface flattening and spiral deformities (Figure 4a). Furthermore, the binarized cross-section images obtained from the micro-CT scan exhibited external surface irregularities, which prevented the creation of a perfect 3D virtual model of the actual instrument (Figure 4b).

### 3.4. Applications

Despite no apparent changes in the overall geometry of the R25 instrument observed under the operative microscope after preparing eight root canals, the employed methodology enabled the superimposition of its 3D models obtained before and after preparation, revealing a 76.11% surface match within a range of ±5 µm. Notably, the most significant geometric modification (≥20 µm) was found at the tip of the instrument, the area that experiences the highest load during root canal instrumentation (Figure 5). Based on the FEA simulation, induced stresses increased from the region of application of the torsional moment to a maximum value of 500 MPa, observed in the apical section. At the same time, the stress distribution in the model’s homogeneous section, located nearest to the point of application of the torsional force, showed values of approximately 50 MPa, followed by a subsequent increase in stress in areas of variable instrument sections, until reaching the critical zone (500 MPa) where fractures occur (Figure 6). These results indicate that the stress distribution followed the principles of Solid Mechanics theory [14]. Lastly, using dedicated software, virtual 3D models obtained through 12 MP optical scanning were utilized to produce images and videos suitable for commercial or educational purposes (Figure 7).

## 4. Discussion

This paper presents a new 3D surface method that utilizes a high-resolution laboratory-based optical scanner to generate accurate virtual models of nickel–titanium (NiTi) instruments, which can be used for various applications in research and education. Over the years, digital technologies have been rapidly evolving in clinical practice with the introduction of digital radiography in the 1980s being just the beginning. Other digital resources, including cone-beam computed tomography, dynamic surgical navigation, CAD/CAM systems, and intra-oral and laboratory-based optical scanners, have been developed to aid in clinical practice [15]. In the research field, new digital resources have also emerged such as micro-computed tomography [16], computational fluid dynamics [17], and the finite elements method [18]. Furthermore, e-learning and teleconsultation initiatives have facilitated better interaction with students and patients [19]. All of these developments point towards a digital era in dentistry [15].

Laboratory-based optical scanners are highly accurate and reliable instruments used to generate virtual models of real objects with an accuracy range of 6 to 50 µm [20]. In the present study, the reliability of this method was first validated by qualitatively comparing NiTi instruments with small tip diameters (ProTaper S1 Gold: 0.18 mm; ProGlider: 0.16 mm) and deformed active blades using both 3D surface scanning and SEM methods. The results showed that the virtual models’ geometry perfectly matched the images obtained from SEM (Figure 2a). The second validation method involved comparing the measurements of virtual cross-sections acquired from NiTi instruments scanned at 12 MP resolution with their corresponding SEM images. Differences ranged from 0.0002 to 0.0182 mm (Figure 2b–e), which were negligible and closer to the lower limit of expected measuring deviations reported by the manufacturer [21]. The final validation approach was to evaluate discrepancies observed when measuring distances between the measuring lines of two virtual instruments and compare those values with the ones reported by the manufacturers (Figure 2f–i). While small differences ranging from 0.0016 to 0.0972 mm were observed, they were higher than the ones obtained when comparing virtual cross-sections with SEM images. This can be explained by the fact that the distance measurements of measuring lines were based on manufacturers’ information, which, although not confirmed in the present study, are dependent on quality control and may vary [22]. Therefore, considering the minimal differences observed in both qualitative and quantitative evaluations between assessment methods, a laboratory-based optical 3D scanner with 12 MP resolution can be considered a reliable and precise tool for performing measurements and creating virtual models of real instruments. In fact, the high precision of the scanner used in the present study is currently considered the gold standard for this type of digital resource [23].

After confirming the reliability of the method using qualitative and quantitative approaches, the next step was to assess the reproducibility of measurements performed at specific levels of three different virtual instruments. To accomplish this, 17 virtual planes were created at the active blade of the 3D models and analyzed for 2D (perimeter, area, long axis, and core diameter) and 3D (surface area and volume) parameters at D1, D8, and D16 levels in two different time frames (Table 1 and Table 2). The measurements of surface area and volume of the entire blade, as well as the 2D parameters, were repeated 10 times and showed very low standard deviation, indicating high precision (Table 1). Additionally, the interclass correlation coefficients were greater than 0.999, demonstrating a perfect reliability between measurements and confirming the reproducibility of the method. This can be attributed to the use of a computer-based automated protocol, which significantly reduces the subjective influence of the operator.

An additional analysis was conducted to compare the accuracy of 3D virtual models of the same instrument obtained through high (12 MP) and low (5 MP) resolution optical scanners with a micro-CT imaging system. Previous research has suggested that the resolution of laboratory-based 3D scanners is a determining factor in their accuracy [23]. As expected, the low-resolution scanner was not able to accurately replicate the instrument’s geometry, whereas the best results were achieved with the 12 MP scanner (Figure 4). Interestingly, the 3D model created by the 12 MP scanner had better quality than the one obtained by scanning the same instrument in a micro-CT device. This difference can be attributed to the variations in the process of acquiring 3D models in each method. While 3D optical scanning is a non-contact method that captures the target object’s three-dimensional shape using a projected light source and measures the displacement of the lines to construct the 3D model [24], micro-CT imaging relies on X-ray scans to create a sequence of 2D slices, commonly known as tomograms, that can be reconstructed using specific algorithms to produce a 3D model of the object without causing any damage. The reconstructed images consist of 256 grayscale levels, which require a segmentation threshold (binarization) to analyze objects through dedicated software. The binarization involves assigning black/white pixel values by imposing explicit cut-off values to the grayscale data. This allows for the grouping of voxels with similar grayscale intensities into spatially meaningful regions, resulting in the creation of a 3D model using specific plugins [25].

Computed tomography can produce artifacts in images, particularly when imaging high-density objects such as NiTi instruments. This is because when an X-ray beam passes through an object composed of photons with different energies it becomes “harder” as the lower-energy photons are absorbed more rapidly than the higher-energy ones. Therefore, the X-rays through the middle of a uniform object are more hardened than those passing through its edges, as they pass through more material [26]. This beam hardening effect causes a higher density halo around an absorbing object in the surrounding space (Figure 4b), which can affect the model rendering and partially explain the irregularities observed at the edges of the instrument after reconstruction. As a result, it may not be possible to obtain a completely reliable 3D virtual model (Figure 4). Additionally, it is possible that the surface irregularities observed in the micro-CT 3D model were primarily due to the limitations of the surface rendering algorithm available in the CTAn software (Bruker-MicroCT, Kontich, Belgium). Future studies may explore the use of alternative software with a wider range of algorithms to improve the quality of the model. It is worth noting that the 3D surface optical scanner is not influenced by the density of objects, as observed in the micro-CT method. Furthermore, compared to other approaches such as optical microscopy [27,28], scanning electron microscopy [29], or size specifications provided by manufacturers [30], 3D optical scanners have been considered more reliable in generating virtual models [27,28,29,30]. Our research confirms that the specific protocols used with micro-CT and 5 MP scanner devices do not produce the same level of precision as the 12 MP scanner and, therefore, are not recommended for reproducing NiTi instruments for research or educational purposes that demand high precision.

The present study demonstrated the research applicability of the surface scanning method by firstly showing the possibility of superimposing two STL volumes of the same instrument acquired before and after the enlargement of eight root canals. A best-fit software calculation was used to compare their dissimilarities, following a widely explored concept when addressing canal shaping using micro-CT technology [31]. This test showed that the scanner is capable of detecting small geometric differences between the two STL volumes, as a permanent deformation (changes >20 µm) was observed at the apical portion of the instrument after preparation (Figure 5), an alteration that was not detected under the dental operating microscope. Therefore, this method can be successfully used to detect small plastic deformations of the NiTi alloy and to compare the overall geometry of different instruments. The second application involves the creation of highly precise virtual models of real instruments to be used in FEA (Figure 6), which can be considered a major advancement in in silico instrument testing methodology. Previous studies have used virtual models obtained by using micro-CT scanning for FEA [18,32]; however, the 3D model created by the 12 MP scanner proposed in this study exhibits superior quality compared to micro-CT (Figure 4). Additionally, the creation of 3D models using micro-CT is an expensive and time-consuming process that demands a higher learning curve than optical scanners. Using dedicated software, STL models obtained by using the proposed method were successfully used to create images (Figure 7) and videos of different NiTi instruments that can be applied for either commercial or educational purposes.

The novelty of the methodology used in this study presented a limitation in the comparison of results to previous studies, as there are no available studies using laboratory-based optical scanners to generate virtual endodontic instruments for reference. Another limitation of the study is the use of only 5- and 12-megapixel-resolution scanners, and it is yet to be determined if scanners with different resolutions, or different brands, would yield similar results, which calls for further research. Future approaches should also focus on other accurately designed experiments following specific goals. These further approaches will help to understand the limitations and true potential of the methodology. The main strength of this study was the ability to produce high-quality virtual models of NiTi instruments with reproducible measurements, which have the potential to become the gold standard for research and teaching purposes in the near future.

## 5. Conclusions

The use of a high-resolution laboratory-based optical scanner enabled the creation of accurate and precise virtual models of various NiTi instruments with potential applications in another related field.

## Figures and Tables

**Table 1:** The cross-section created by 17 virtual planes positioned from D0 to D16 in the virtual instrument were analyzed regarding 2D parameters of perimeter (mm), area (mm2), long axis (mm), and core diameter (mm). Calculation of 2D parameters was repeated 10 times at D1, D8, and D16 levels to calculate the standard deviation of the results. In each instrument, measurements were repeated twice in an 8-week interval and their similarity was calculated using the interclass correlation coefficient (ICC) test.

Levels	Reciproc R25				ProFile Size 25, Taper 0.06				ProTaper Next X2			
	Perimeter	Area	Long Axis	Core	Perimeter	Area	Long Axis	Core	Perimeter	Area	Long Axis	Core
	**Measurement assessment (average results after 2 evaluations)**											
D0	0.6367	0.0306	0.2236	0.1706	0.6835	0.0294	0.2262	0.1548	0.5733	0.0254	0.2323	0.1633
D1	0.9107	0.0621	0.3209	0.2350	0.8681	0.0475	0.2819	0.1922	0.7654	0.0388	0.2638	0.1766
D2	1.1714	0.1031	0.4167	0.3087	1.0226	0.0658	0.3292	0.2259	0.9116	0.0564	0.2992	0.2047
D3	1.3553	0.1328	0.4943	0.3282	1.2056	0.0901	0.3893	0.2635	1.0722	0.0757	0.3566	0.2350
D4	1.4951	0.1625	0.5307	0.3612	1.3686	0.1133	0.4499	0.2894	1.2156	0.0987	0.4321	0.2727
D5	1.6439	0.1955	0.5925	0.4041	1.5287	0.1402	0.5006	0.3228	1.4141	0.1321	0.4926	0.3154
D6	1.7946	0.2292	0.6529	0.4197	1.6940	0.1710	0.5594	0.3562	1.5791	0.1631	0.5476	0.3522
D7	1.9273	0.2593	0.7231	0.4443	1.8697	0.2028	0.6117	0.3848	1.7345	0.1959	0.6089	0.3807
D8	2.0304	0.2878	0.7435	0.4638	2.0353	0.2407	0.6656	0.4177	1.8997	0.2335	0.6719	0.4158
D9	2.1249	0.3088	0.8031	0.4689	2.2079	0.2784	0.7126	0.4461	2.0626	0.2736	0.7359	0.4485
D10	2.2118	0.3304	0.8438	0.4775	2.3800	0.3208	0.7785	0.4764	2.2257	0.3174	0.7687	0.4809
D11	2.2770	0.3450	0.8697	0.4814	2.5555	0.3694	0.8394	0.5088	2.3857	0.3650	0.8458	0.5164
D12	2.3388	0.3584	0.9118	0.4815	2.7120	0.4158	0.8807	0.5381	2.5406	0.4124	0.9001	0.5483
D13	2.3898	0.3701	0.9454	0.4824	2.8849	0.4699	0.9334	0.5690	2.6602	0.4509	0.9516	0.5713
D14	2.4380	0.3811	0.9647	0.4836	3.0431	0.5201	0.9901	0.5971	2.7733	0.4870	0.9864	0.5932
D15	2.4881	0.3916	0.9995	0.4838	3.1878	0.5775	1.0317	0.6362	2.8482	0.5142	1.0130	0.6156
D16	2.7687	0.5421	1.0935	0.6669	3.3550	0.6341	1.0866	0.6382	3.0443	0.6013	1.0576	0.6714
	**Standard deviation (after performing 10 measurements of each parameter)**											
D1	0.0044	0.0019	0.0038	0.0003	0.0112	0.0006	0.0062	0.0004	0.0059	0.0028	0.0047	0.0007
D8	0.0020	0.0187	0.0191	0.0003	0.0036	0.0015	0.0049	0.0007	0.0023	0.0006	0.0041	0.0002
D16	0.0092	0.0044	0.0063	0.0021	0.0087	0.0007	0.0228	0.0013	0.0156	0.0078	0.0133	0.0029
	**Interclass correlation coefficient (comparing results after 2 evaluations)**											
	1.000	1.000	1.000	1.000	1.000	1.000	1.000	1.000	1.000	0.999	0.999	0.999

**Table 2:** 3D parameters of surface area (mm^2^) and volume (mm^3^) calculated for the whole active blade (from D0 to D16) of 3 NiTi instruments. Calculation of 3D parameters was repeated 10 times to calculate the standard deviation of the results.

Levels	Reciproc R25		ProFile Size 25, Taper 0.06		ProTaper Next X2	
	Volume	Surface Area	Volume	Surface Area	Volume	Surface Area
	**Measurement assessment (average results after 2 evaluations)**					
D0 to D16	4.178	30.635	4.351	36.446	4.055	30.263
	**Standard deviation (after performing 10 measurements of each parameter)**					
D0 to D16	0.0113	0.0576	0.0226	0.1446	0.0244	0.3098

## Data Availability

Data available on request from the authors.
